# Synovial Extracellular Vesicles: Structure and Role in Synovial Fluid Tribological Performances

**DOI:** 10.3390/ijms231911998

**Published:** 2022-10-09

**Authors:** Layth Ben-Trad, Constantin Ionut Matei, Mirela Maria Sava, Samira Filali, Marie-Eve Duclos, Yves Berthier, Michel Guichardant, Nathalie Bernoud-Hubac, Ofelia Maniti, Ahmed Landoulsi, Marie-Genevieve Blanchin, Pierre Miossec, Thierry Granjon, Ana-Maria Trunfio-Sfarghiu

**Affiliations:** 1 Laboratory of Contact and Structural Mechanics, University of Lyon, CNRS, INSA Lyon, UMR5259, Villeurbanne, 69100 Lyon, France; 2 Institute de Chimie et Biochimie Moléculaires et Supramoléculaires, ICBMS UMR 5246, University of Lyon, Université Lyon 1, CNRS, 69622 Lyon, France; 3 Faculty of Sciences of Bizerte, University of Carthage, Laboratory of Risques Liés aux Stress Environnementaux: Lutte et Prévention, Zarzouna 1054, Tunisia; 4 Institut Multidisciplinaire de Biochimie des Lipides, 69621 Villeurbanne, France; 5 Institute Lumiere Mat, University of Lyon, CNRS, UCBL, ILM, UMR5506, 69622 Villeurbanne, France; 6 Unit of Immunogenetics & Inflammation EA-4130 & Department of Clinical Immunology and Rheumatology, University of Lyon, Hôpital Edouard Herriot, 69437 Lyon, France; 7 Charles River Laboratories, 13, Allée de Nudlingen, 27950 Saint-Marcel, France

**Keywords:** synovial fluid, lubricant, rheumatoid arthritis, osteoarthritis, nanoparticles, lipid structures

## Abstract

The quality of the lubricant between cartilaginous joint surfaces impacts the joint’s mechanistic properties. In this study, we define the biochemical, ultrastructural, and tribological signatures of synovial fluids (SF) from patients with degenerative (osteoarthritis-OA) or inflammatory (rheumatoid arthritis-RA) joint pathologies in comparison with SF from healthy subjects. Phospholipid (PL) concentration in SF increased in pathological contexts, but the proportion PL relative to the overall lipids decreased. Subtle changes in PL chain composition were attributed to the inflammatory state. Transmission electron microscopy showed the occurrence of large multilamellar synovial extracellular vesicles (EV) filled with glycoprotein gel in healthy subjects. Synovial extracellular vesicle structure was altered in SF from OA and RA patients. RA samples systematically showed lower viscosity than healthy samples under a hydrodynamic lubricating regimen whereas OA samples showed higher viscosity. In turn, under a boundary regimen, cartilage surfaces in both pathological situations showed high wear and friction coefficients. Thus, we found a difference in the biochemical, tribological, and ultrastructural properties of synovial fluid in healthy people and patients with osteoarthritis and arthritis of the joints, and that large, multilamellar vesicles are essential for good boundary lubrication by ensuring a ball-bearing effect and limiting the destruction of lipid layers at the cartilage surface.

## 1. Introduction

Synovial articulations exhibit extremely low levels of friction under high physiological pressures (around 10 MPa) [1] and can thus sustain a wide variety of movements with an exceptional lifetime of up to 80 years. These remarkable performances are linked to the tribological triplet: cartilage, synovial fluid (SF), and the musculo-ligamentous-joint system. The triplet has two essential functions: (1) to allow any movement controlled by the motor endplate of the musculo-ligamentous-joint system; and (2) to ensure an optimal load transmission with maximum damping (cartilage) and minimum friction (synovial fluid). Synovial joints are subjected to a varying load associated with different speeds in the course of their function.

Much work has been done to explain the mechanisms of joint lubrication, establish the nature of the interactions between the cartilage and the SF, and explain the SF’s exceptional tribological behavior [2,3,4,5,6,7]. SF is a thick liquid that lubricates the articular surfaces and ensures their smooth movement against each other by preventing direct friction; thus, it prevents wear of the joints. The different phases of support have been described by theoretical models including hydrodynamic, boundary, weeping, elasto-hydro-dynamic, squeeze film, or boosted regimes. All these models suppose that the cartilaginous surfaces are separated by a continuous full-fluid lubricating film of a few ten to a few hundred nanometers in thickness [8,9,10]. In this case, the bulk viscosity of the fluid is of utmost importance, and it is mainly ensured by hyaluronan (HA) [5,11,12,13], a linear polysaccharide abundant in the joint. However, these lubrication models do not fully explain the mechanisms of the joint lubrication and it is likely that a mixed regime operates including both fluid-film and boundary lubrication [5]. In the latter case, the nature of the boundary layer at the cartilage surface gains particular importance [5,14,15]. Thus, it was concluded that both bulk SF composition and the elements of interaction at the cartilage level have relevance in the analysis of SF lubrication.

In 1972, Swann identified a proteoglycan-type molecule at the cartilaginous surfaces [16], thereby opening the way for further research on joint boundary lubrication. Several tribological tests performed with samples of this molecular component of the synovial fluid revealed low friction coefficients attributed to its lubricant properties, hence its name “lubricin” [17,18]. However, lubricin [19] or other cartilage macromolecules [20,21] are not capable either by themselves or in surface layers of reducing sliding friction coefficients to the very low levels (0.001) reported for the major synovial joints [22].

Lipids, either spread as bilayers at the cartilage surface [23,24,25] or as vesicles in the SF [26,27,28,29] have been invoked as being responsible for the remarkable lubrication properties of SF joints. Several studies showed the presence of discontinuities in the lubricating film, and microvesicles of a few μm in diameter were identified in samples of rat synovial fluid [30,31,32]. Interestingly, the SF micro vesicles corresponding to such film discontinuities were larger than the thickness of the lubricant film estimated by hydrodynamic models [33].

Kawano et al., in 2003, examined the effects in vivo of a mixture of high molecular weight HA and liposomes on joint lubrication and articular cartilage degeneration on a rabbit OA model and showed that a liposomes/HA mixture reduces friction coefficients and better protects cartilage surfaces [34]. These conclusions were reinforced by the report of Forsey et al. in 2006, which measured the ability of HA, Dipalmitoylphosphatidylcholine (DPPC), and mixtures of HA and DPPC to reduce start-up friction in an OA-damaged human cartilage model [35].

It has therefore been proposed that HA and PL work in concert for ideal lubrication properties [36]. Interestingly, extracellular lipid vesicles present in SF are not classical unilamellar vesicles, but multilayer ones interacting with each other and forming supramolecular networks [27,37]. Such supramolecular networks have a remarkable impact on the overall rheology of the lubricant film [6,38].

Despite the progress made in physicochemical characterization techniques and models of boundary lubrication, the manner in which PL influence friction is still insufficiently understood. Are EV present in SF as multilayer lipid structures responsible for the exceptional physical properties of synovial joints? Do they constitute a potential biomarker for joint pathologies?

Several studies have analyzed the lipid composition of SF in physiological and pathological conditions. For instance, using several separation methods, 12 types of lipids in synovial EV including cholesterol esters, ceramides, triacylglycerols, diacylglycerols, free fatty acids, and PL were detected and quantified [39]. The dominant PL classes in SF are the ones that contain choline Phosphatidylcholine (PC), Sphingomyelin (SM), and (Lysophosphatidylcholine (LPC)) along with other PL classes in smaller amounts [40,41]. Compared with control SF, OA-SF both in human and canine [42] models have significantly higher levels of total lipids and higher lipid diversity, including PC, LPC, phosphatidylinositol (PI), and SM. Alterations in individual levels of PL are regularly reported but it is difficult to propose a general hallmark in the lipid profile of SF for OA or RA pathologies [41,43,44,45].

The lubricant capacity of lipid vesicles under ex vivo conditions depends on their ultrastructure in addition to their biochemical composition. Large multilamellar vesicles (MLV), composed of saturated lipids, such as 1,2-dimyristoyl-sn-glycero-3-phosphocholine (DMPC), or 1,2- dipalmitoyl-sn-glycero-3-phosphocholine (DPPC) are superior lubricants in comparison to MLV composed of unsaturated phosphatidylcholines [46,47]. Introducing cholesterol into liposomes results in less effective lubricants [46]. MLVs are better lubricants than small unilamellar vesicles [48,49] Goldberg et al. demonstrated that layers of PC liposomes attached to cartilage surfaces reduce the coefficient of friction between them to unprecedentedly low values [28].

Moreover, synovial vesicles obtained from animal SF samples have been shown to be constituted of several lipid bilayers (multilamellar assemblies) containing pellets of HA and proteins [7,26]. We have previously described these extracellular vesicle assemblies as a gel-in vesicular structure, which showed remarkable lubricating capacities in terms of friction coefficient and wear. In contrast, a gel-out situation, in which HA and proteins are present in the external medium, outside of the vesicles, showed weaker lubricant qualities [7].

In this report, we aim to perform a biochemical, tribological, and ultrastructural characterization of SF from healthy, OA, and RA patients in order to associate biochemical signatures with vesicle ultrastructure and lubricant capacities. The lipid composition of the different SFs was analyzed with an emphasis on PL, which are the major/determining components of lipid bilayers. We then tried to connect our findings with the lubricating abilities of the synovial fluid.

We found a difference in the biochemical, tribological, and ultrastructural properties of the synovial fluid in healthy people and patients with osteoarthritis and arthritis of joints. Alterations in lipid composition were observed, with an increase in the amount of lipids in the event of joint disease, and a decrease in the ratio of PL to total lipids, concomitant with micro-vesicle de-structuration to different extents. The occurrence MLV filled with HA and protein-containing gel (gel-in status) conferred excellent tribological properties to healthy SF, with a good lubricating capacity and full preservation of the surface-coated lipid multilayers in friction tests. On the contrary, small size vesicles with extravesicular glycoprotein gel (gel-out status) were observed in OA and RA samples, resulting in poor lubricating and nanomechanical properties, with the destruction of the lipid multilayer interface in friction tests. Biochemical composition, vesicle structure, and lubricating capacities are therefore intimately linked in SF and should be considered together to improve the diagnosis and prognosis of joint diseases or to develop new therapeutic strategies.

## 2. Results and Discussion

### 2.1. Lipidomic Analysis of Human and Dog Synovial Fluid under Non-Pathological Conditions

**Figure 1:**
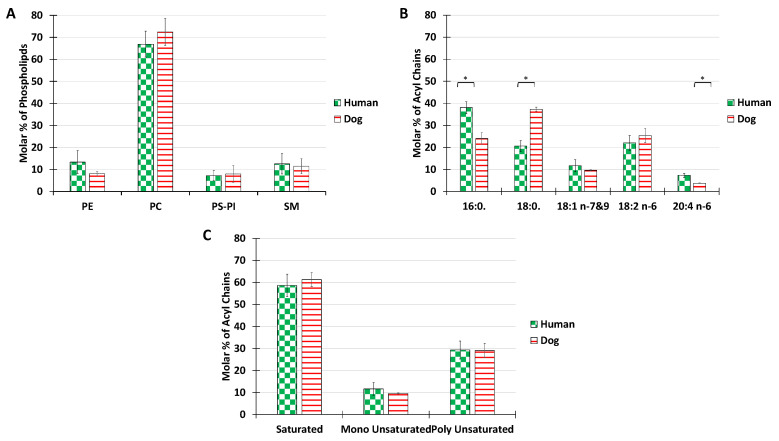
Lipid composition of SF from the knee joints of dogs and human healthy volunteers. The molar percentage of phospholipid classes in the total PL extract (**A**), acyl chain distribution in the total PL extract (**B**), and molar percentage of saturated, monounsaturated, and polyunsaturated acyls present in the total PL extract (**C**). Lipids were quantified as described in Materials and Methods. Data are presented as the mean ± SD of 5 dogs and 7 humans. *p*-values (*t*-test) less than 0.05 were considered significant (*).

PL are the major constituents of membrane bilayers in the mammalian cell. Therefore, in the first series of measurements, we analyzed the distribution of phospholipid classes extracted from the synovial fluid of humans and dogs with no osteoarticular pathologies (Figure 1B).

The main choline-based PL for both dogs and human were phosphatidylcholine (68%) and sphingomyelin 12%. The overall distribution of PL classes was roughly maintained for the two species. The percentage of PE reached 13% in humans and 8% in dogs. The percentage of negatively charged lipids, PS and PI confounded, was below 11% for both species. PE, PS, or PI, abundant in other membrane structures such as plasma or ER membranes, where they are involved in active processes such as membrane trafficking or signaling, were only minor components in SF. The predominant phospholipids PC and SM are known to form stable bilayers. The PL composition of SF is thus consistent with the mechanical role of synovial vesicles, for which stable bilayers are required. Our results are in agreement with those of Kosinska et al., 2016, who showed that human SF and canine SF had comparable lipid compositions, with choline-containing phospholipids being the major class [42].

The acyl chain distribution was investigated (Figure 1B). In both humans and dogs, only 5 acyl chain species were present above the limits of detection, in contrast with the much higher diversity recorded in cellular membranes [50]. The most abundant species was palmitoyl in humans and stearoyl in dogs. In terms of unsaturated fatty acids, linoleic acid was predominant in both species, but some differences were recorded in the proportion of arachidonic acid, with a higher ratio in humans. A possible reason for the differences in the acyl compositions of the synovial joints might be the different nutritional behavior between humans and dogs. Indeed, the high consumption by humans of palmitic acid, which is found in a large number of foods, may be responsible for a large amount of the palmitate measured. Similarly, the more animal products consumed in the diet, the greater the intake of long-chain polyunsaturated fatty acids such as arachidonic acid (AA) (20:4) (Abedi & Sahari, 2014; Mann et al., 2006), and dogs are generally bigger meat eaters than humans.

Despite these nutrition-related differences, overall, both species showed similar global proportions of saturated, monounsaturated, or polyunsaturated fatty acids with a consistent ratio of 6:1:3 (Figure 1C).

### 2.2. Lipidomic Analysis of Human Synovial Fluid under Non-Pathological and Pathological Conditions

**Figure 2:**
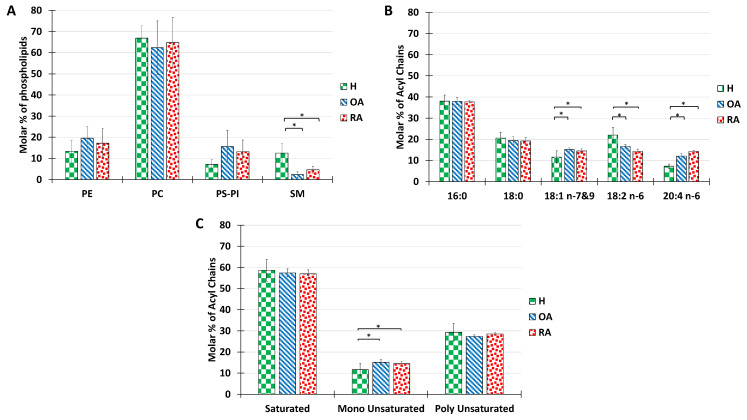
Lipid composition of SF from the knee joints of human healthy volunteers and OA or RA patients. The molar percentage of phospholipid classes in the total PL extract (**A**), acyl chain distribution in the total PL extract (**B**), and molar percentage of saturated, monounsaturated, and polyunsaturated acyls present in the total PL extract (**C**). Lipids were quantified as described in Materials and Methods. Data are presented as mean ± SD of 7 healthy, 3 OA, and 3 RA humans. *p*-values (*t*-test) less than 0.05 were considered significant (*).

We then investigated whether this overall composition, which seems to be conserved between omnivore species, may be altered by pathological situations. To answer this question, the PL composition of healthy (H), OA, and RA synovial fluid was analyzed in detail (Figure 2). Our results indicate that the overall distribution of PL classes was maintained, except for a significant decrease of sphingomyelin observed between healthy samples and both pathologies (Figure 2A). As a bioactive lipid, sphingomyelin is implicated in a variety of physiological functions such as inflammation, once hydrolyzed to ceramide by the enzyme sphingomyelinase [51]. Metabolites obtained from ceramide such as S1P have key roles in the regulation of the trafficking and the functions of immune cells. S1P drives the differentiation of many immune cells, inducing changes in their function and regulating the production of pro-inflammatory cytokines [52].

The analysis of acyl chains in humans revealed that both RA and OA samples showed a majority of palmitoyl chains in their composition, followed by stearoyl, with no significant difference relative to healthy controls (Figure 2B). Significant differences were recorded in the proportions of unsaturated acyl chains with a slight increase in oleic acid (C18:1), a decrease in linoleic acid (C18:2), and an increase in arachidonic acid (C20:4). (C18:2) is the precursor of the (C20:4), which in turn is involved in inflammatory processes as a precursor of inflammatory factors. Therefore, we can relate the increase in C20:4 percentage to inflammatory processes occurring in the joint.

The distribution of acyl chains according to the degree of saturation shows no significant difference between healthy and pathological synovial fluids. The above-mentioned ratio of 6:1:3 was roughly maintained for all samples tested (Figure 2C)

Despite some differences in SM or polyunsaturated acid proportion, most probably due to inflammation processes, the differences in PL characteristics do not explain the dramatic decrease in lubricating capacities described in RA and OA pathologies. Therefore, we searched for additional alterations in other SF components.

### 2.3. Alterations in Other SF Components

**Figure 3:**
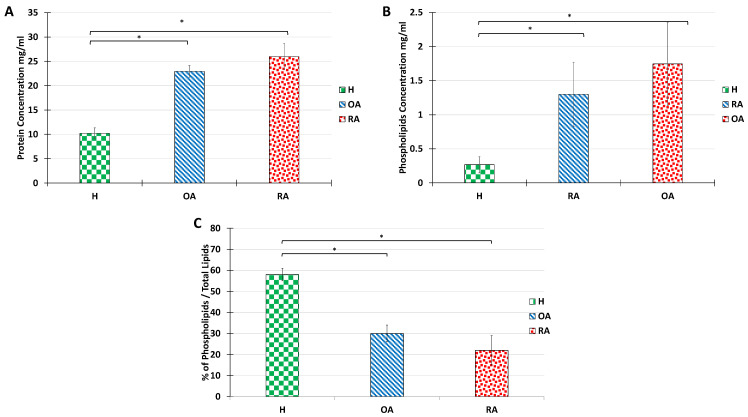
Alterations in protein and total lipid concentration in SF from the knee joints of human healthy volunteers and OA or RA patients. Protein concentration (**A**), PL total amount (**B**), and PL percentage are calculated as the ratio of PL acyl chain molar concentration over total lipid acyl chain concentration (**C**). Data are presented as mean ± SD of 7 healthy, 3 OA, and 3 RA humans. *p*-values (*t*-test) less than 0.05 were considered significant (*).

Mazzucco et al. have previously described that pathologies such as OA and RA, analyzed here, induce an increase in PL and protein amount, with a remarkable abundance of immunoglobulins [4]. Thus, we evaluated the amount of protein present in the SF extracts using a Bradford test (Figure 3A). The protein amount in healthy samples was estimated at 10 mg/mL, whereas we observed an over 2-fold increase in OA samples and a 2.5-fold increase in RA samples were recorded. The increase in protein content is in line with previous reports for OA pathologies [4], but is more limited than those reported for RA. The major difference observed in our case can be explained by the fact that RA samples analyzed here were obtained from patients with incipient pathology, in which SF was not contaminated with blood, and therefore protein amounts in samples were below the literature data, which ranges between 36 mg and 54 mg/mL. The protein profile for a healthy patient was dominated by albumins, whereas for RA and OA, the protein profile was dominated by globulins, which is consistent with a high inflammatory profile (not shown).

The total PL content in healthy, OA, and RA synovial fluids was also measured (Figure 3B). In line with previous reports [4,42], PL content increased over 10 folds from 0.1–0.2 mg/mL in healthy situations to 1 and 2 mg/mL for OA and RA, respectively.

Yet, it is important to note that the overall lipid amount in SF also increases in pathological situations. Indeed, neutral lipids, including cholesterol esters and glycerides are very abundant in OA and RA synovial fluids. Therefore, although PL amounts increase in OA and RA situations, the PL/neutral lipids ratio decreases drastically (Figure 3C). It is known that when neutral lipids are highly abundant, the bilayer structure of the vesicle is no longer stable. Therefore, we investigated whether the ultrastructure of the synovial vesicles could be maintained with the increase in the proportion of neutral lipids.

### 2.4. Ultrastructural Analysis of Human Synovial Joint

**Figure 4:**
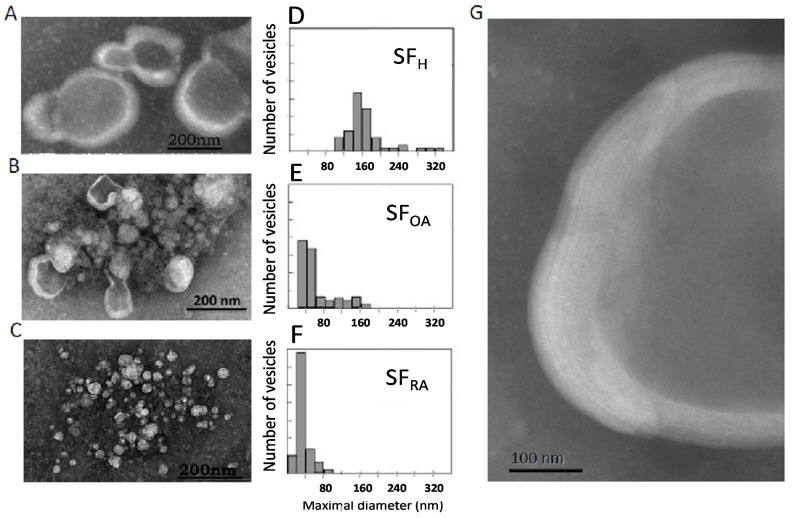
Ultrastructure of representative samples of normal and pathological SF: Transmission electron microscopy of SF healthy donors (**A**) or SF from patients with OA (**B**) or RA (**C**). Size distribution of vesicles present in healthy SF samples (**D**), OA SF samples (**E**), or RA SF samples (**F**); Multilamellar structures characteristic of healthy SF samples in a 5 times enlargement of figure A (**G**). Size distribution analysis: (**D**) Min-Max: 100–556 nm; Med ± SD 157 ± 6 nm; (**E**) Min- Max: 30–171 nm; Med ± SD: 45 ± 5 nm (**F**) Min-Max: 30–171 nm; Med ± SD: 45 ± 5 nm.

The structural elements present in the different types of SF were analyzed by transmission electron microscopy (TEM) with negative staining. Three types of TEM patterns were distinguished (Figure 4), depending on the pathological state of the patient.

The ultrastructure of healthy synovial fluid showed a majority of MLV with a size around 180 nm (Figure 4A,D). Higher magnification showed a multilamellar structure of the lipid envelope (Figure 4G) with 5 to 7 lipid bilayers. Depending on the viscosity of the aqueous medium in which the dye is present, there is a lighter gray shade (negative stain diffuses less in a more viscous medium) or an intense gray shade (negative stain diffuses more in a more fluid medium). The vesicle inner core appeared as a light grey medium, whereas the background separating the vesicles was darker than the vesicle center. We can conclude that the viscous HA and protein-containing gel are embedded inside the MLV. This structure is conserved among animal species [26,27] and was successfully reproduced ex-vivo and described as “gel-in” type vesicles [7,26].

We suggest that this structure is a hallmark of healthy, physiological SF. We further checked if this structure was altered in pathological conditions and if the excellent lubricating properties of SF depended on the multivesicular structures described above.

Interestingly, in samples obtained from chronic OA patients, a mixture of small unilamellar and large multilamellar vesicles was observed (Figure 4B,E), mainly ranging from 30 to 170 nm, whereas RA samples only showed small unilamellar vesicles of around 40 nm (Figure 4C,F). These structural modifications can be linked strongly to changes in lipid composition described above. First, the decrease in the percentage of the bilayer-forming lipid SM may contribute to the loss of lipids’ ability to form multilayer structures. However, such modifications, although significant, were rather small and may not fully explain such a drastic effect on vesicle ultrastructure. Second, a very strong decrease in the PL/neutral lipid ratio was evidenced (Figure 3C). This strong decrease definitively alters the stability of lipid bilayers and results in the formation of small vesicles or micelles.

Large, multilamellar vesicles were therefore characteristic of normal SF, whereas the occurrence of a mixture of large and small vesicles for OA samples and small vesicles for RA samples would be the signature of the joint inflammatory status.

In OA, SF lipid structures were surrounded by a clear background. This indicated the presence of a high concentration of HA not encapsulated in vesicles that engenders a very viscous medium, also previously reported in the case of “gel-out” biomimetic fluids [7,26]. In RA SF, a dark background was observed outside the vesicles, indicating an aqueous medium.

### 2.5. Rheological and Tribological Analysis of SF Lubricating Properties

**Figure 5:**
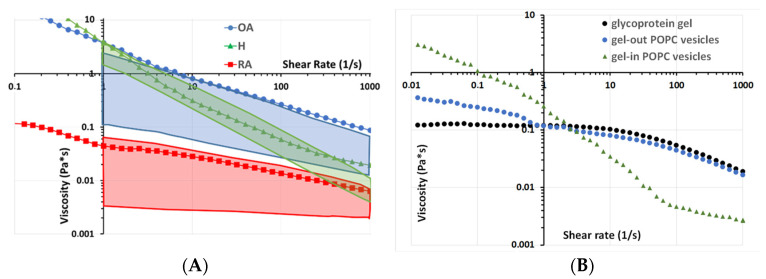
Rheological analysis: Lubricant viscosity as a function of the shear rate measured in a cone/plane configuration under shear stress. (**A**) SF from normal and pathological sources. (**B**) Biomimetic vesicular structures.

Rheological and tribological analyses were performed to check if the observed ultrastructure alteration may affect the well-known rheological and tribological properties of synovial fluid. Several modes of lubrication are relevant in synovial joints: In the hydrodynamic mode or fluid film lubrication, a wedge of fluid forms such that surface movement squeezes fluid from the base of the wedge to its apex. The viscosity of the fluid, as the key parameter of hydrodynamic lubrification mode, was measured in different pathophysiological situations, as a function of shear stress and compared with literature data (Figure 5A).

Our results indicate that healthy SF displayed a non-Newtonian behavior: viscosity decreased with shear rate (Figure 5A, green triangles), which is in line with previously published data [53] (Figure 5, green box). This behavior is generally explained by changes in the structural organization of HA present in the SF upon increasing shear rate.

The pathological synovial fluid samples also displayed shear-thinning behavior, as indicated by the decrease in viscosity with increasing shear rate (Figure 5A). These decreases were more important in OA than in RA samples.

The viscosity of H and OA appears to be almost the same at a shear rate of 2 s^−1^ (which corresponds to a walking cycle speed). The difference starts to grow at 10 s^−1^ (which corresponds to running speed). This difference tends to stabilize around 0.5 Pa.s above 100 s^−1^ with OA having lower shear-thinning behavior (Figure 5, blue circles), which is also similar to the literature (Figure 5A, blue box). However, up to date, no explanation has been given. Based on the structural data from Figure 4, we propose that this behavior is due to differences in the vesicular structures of the synovial fluid. To validate this hypothesis, we reproduced the “gel-in” and “gel-out” vesicular structures by methods similar to those described in Sava 2013 with the same lipid concentration of 0.3 mg/mL and the same concentration of glycoprotein gel (hyaluronic acid 3 mg/mL and albumin 20 mg/mL) (Figure 5B). Interestingly, the H sample rheological behavior is similar to that of gel-in vesicular structures (Figure 5B, green triangles), whereas the behavior of OA samples is close to that of gel-out vesicles (Figure 5B, light blue circles), which in turn is similar to the one of a glycoprotein gel constituted of HA and proteins (Figure 5B, black circles). Consequently, for OA, the loss of gel-in structure and the presence of HA outside the vesicles may explain the higher difference in viscosity for OA compared with H.

SF from RA patients displayed a lower viscosity value than H and OA over the entire shear rate range analyzed in the experiment (Figure 5A, red squares), in line with previously published studies (Figure 5A, red box).

These studies consider viscosity as the key parameter in lubrication, and the authors conclude that high viscosity solely ensures a good separation between the surfaces in contact. However, the hydrodynamic mode, and liquid viscosity, in particular, do not explain all joint lubrication characteristics, namely low friction, and wear at the cartilage level. Indeed, high viscosity is expected to induce friction and wear at the contact surface. Therefore, a second mode of lubrication is generally considered, i.e., boundary layer lubrication. In boundary lubrication, a component of the lubricant adheres to the articulating surfaces, forming a molecular coating. Because the molecules are adsorbed, rubbing surfaces suffer less damage.

Rheological solicitations do not allow one to analyze the role of the lipid vesicles in lubrication because the experimental gap in rheometer settings (> 0.5 mm) is far greater than the scale of lipid bilayers.

**Figure 6:**
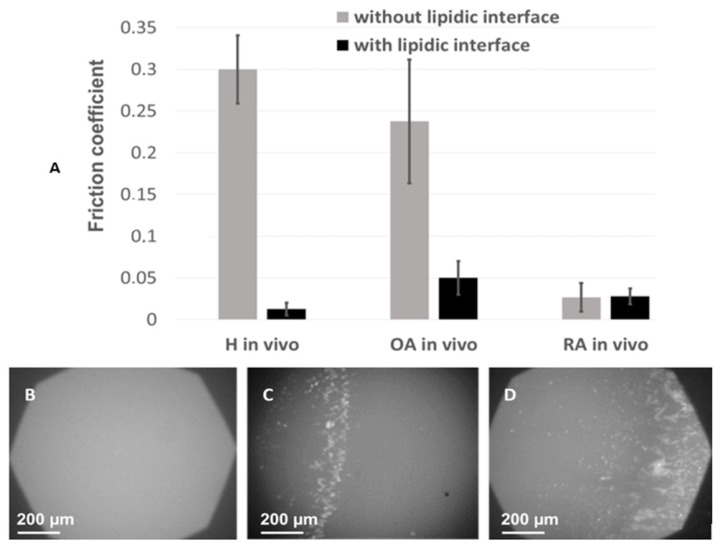
Tribological analyses using Heathy, RA, and OA SF: Fiction coefficient upon rubbing of tribometer plates in the presence (color) or absence (color) of lipid bilayers spread at the surface (lipid interface) obtained with SF of healthy, RA, or OA donors as a lubricant (**A**); Visualization by fluorescence microscopy of the lipid bilayer spread at the plate after friction tests (60-min friction) in the presence of healthy SF (**B**), OA SF (**C**), or RA SF (**D**) between the rubbing plates. NBD-PE was used as a fluorescent probe embedded in the spread lipid bilayer. The destruction of the lipid bilayers is shown by the fragmentation and accumulation of fluorescent material at the edge of the contact zone between the two rigid surfaces. Error bars represent standard deviation (m ± SD; *n* = 3).

In order to show the effect of alterations of vesicle structure on the lubricating properties of SF, the boundary lubrication mode was mimicked in tribological tests performed using our homemade tribometer with rubbing surfaces covered by lipid bilayers doped with fluorescent lipid NBD-PC (Figure 6), as described in Materials and Methods. This experimental set-up reproduces in vivo conditions in the joint, in which the cartilage surface is covered by PL multilayers, and the lubricant between the two surfaces is constituted by the SF.

Samples of synovial fluid were included between the two surfaces and the friction coefficient was measured in the presence or absence of lipid layers, whereas wear of the two rubbing surfaces was analyzed by fluorescence microscopy. The 0.3 friction coefficient measured in the absence of lipids at the rubbing surfaces was high, in line with the high viscosity of SF under non-pathological conditions. It drastically decreased in the presence of lipid bilayers on the rubbing surfaces and in the presence of healthy SF samples (from 0.3 ± 0.03 to 0.008 ± 0.002 (m ± SD; Figure 6). Considering the physiological structure of the joint, where lipid bilayers cover the cartilage [54,55,56], this result is in line with the previously one previously reported one [57], and confirms the physiological role of SF in limiting friction at the joint.

For OA and RA samples, in the presence of lipid bilayers, the friction coefficient was 2.5 to 3 times higher, 0.019 ± 0.012 and 0.024 ± 0.013, respectively (Figure 6). To illustrate an incipient in vivo cartilage wear, the same experiment was repeated in the absence of lipid bilayers at the surface. In this case, the friction coefficient was sensibly higher for OA samples but had almost the same value for RA samples as in the presence of the bilayer. The low friction coefficient observed in the presence of RA SF and in the absence of the lipid bilayers at the rubbing surfaces may be explained by the low viscosity of RA SF, as measured in Figure 5.

Finally, fluorescence images of the lipid bilayers at the rubbing surface, simultaneously recorded during the friction tests (Figure 6), showed a homogeneous surface after rubbing in presence of health SF, implying that the lipid bilayers were not damaged during the friction tests. According to the role of SF in the synovial joint, the simultaneous presence of lipid bilayers at the rubbing surfaces and SF of adequate composition and structure leads to low friction coefficient and absence of wear.

Significant degradation of the lipid bilayer was observed with OA synovial fluids, and this phenomenon was even more pronounced for RA samples (Figure 6).

These results indicate that MLV, characteristic of normal SF, are, therefore, essential for good boundary lubrication. This lubrication effect has been primarily attributed to the hydration layers surrounding the PC groups (up to 20 hydration water molecules) at the outer surface of the opposing vesicle layers as they slide past each other. These hydration water molecules are tenaciously attached, yet mobile, thereby providing a ball-bearing-like effect [22]. Moreover, the encapsulation of HA in lipid vesicles prohibits interaction between the polysaccharide and the PL headgroups at the rubbing surface and diminishes the wear in severe situations of lubrication (limit conditions).

The occurrence of small vesicles, with a mixture of large and small vesicles for OA samples and homogeneous small vesicles for RA samples together with incomplete HA encapsulation drastically diminishes SF lubricant quality in boundary lubrication conditions by increasing friction and wear.

## 3. Conclusions

Understanding the role of synovial vesicles, a very particular type of EV, in joint lubrication and function is not only of utmost importance in our comprehension of joint tribology, but also in improving the diagnosis and treatment of joint diseases.

Until now, most of the research work in synovial lipids was divided into two fields: (1) a medical one, with the very detailed analysis of lipid composition [39,42,43,44,58], which showed an alteration of phospholipids in pathological states, but did not analyze the structure, nor the rheological consequences of such modifications, and (2) a biomimetic field [20,22,29], which is interested in the vesicular structures in relation to the lubrication process. Our work tried to match both fields and has established, directly from healthy and pathological samples, that it is not the intrinsic membrane structures or the lipid composition that matters for the rheology of the synovial fluid, but more the lipids over proteins and PL over total lipids ratios that causes the destructuring of the synovial vesicles, and eventually decreases the lubricating properties of the synovial fluid.

An important finding of the present study was that, by comparing healthy and OA or RA samples, we showed that the PL amount increased in pathological samples, but that the PL/neutral lipid ratio decreased, suggesting destabilization of bilayer structures. Interestingly, overall saturated/monounsaturated/polyunsaturated fatty acid ratio and the overall PL class distribution were not severely altered, but some differences were notable in SM, and in arachidonic acid level, whose effect can logically be related to their key role in the inflammatory pathway in joint pathologies.

As the PL/neutral lipid ratio decreases, vesicle morphology was profoundly changed in samples from OA and RA patients, with a loss of the characteristic multilayer vesicular structure. The morphology of the vesicles depends on the lipid composition of the medium. It has been shown that relatively low PL concentrations (ca. 0.3 mg/mL), such as those found in healthy SF, are sufficient to generate PL multilayers constituted by the stacking of 3 to 7 bilayers [57]. In a pathological context, increasing amounts of non-bilayer forming lipids, surpassing the previously reported increase in PL, may alter this morphology.

In terms of lubrication, our data indicated that the presence of large multilamellar vesicles (200 nm to 2 µm in diameter) conferred excellent tribological properties to SF, which is associated with a good lubricating function and a full resistance to friction forces of the lipid bilayers constituting the interface with the cartilage. These properties confirm those observed in control SFs from healthy animals, e.g., dogs, horses, or rats [26,27], and with liposomes [7], whose excellent tribological properties have been attributed to a ball-bearing like the effect of the highly hydrated phosphocholine groups exposed at the liposome surface [22,28].

Conversely, small size vesicles (20 to 180 nm), as observed in SFs from patients with RA, resulted in poor lubricating and mechanical properties of SF, with the destruction of the lipid bilayer interface during friction tests. This type of effect also applied to SFs collected from patients with OA. Yet, in the absence of lipid layers, the friction coefficient was sensibly lower for RA than OA SF. At a first glance, this finding may suggest better lubricating properties in the RA context than in an OA pathological context. However, it is important to bear in mind that in a full joint, both boundary and hydrodynamic regimens make determinant contributions. As shown in Figure 5, the viscosity of RA SF is low compared with that of OA or healthy control, which means that, on the scale of a real contact, the thickness of the lubricant film will be insufficient to protect against wear. Consequently, friction coefficients in the presence or absence of bilayers have extreme relevance:

In the absence of lipid interface, the high friction coefficients obtained for healthy SF correlate with high sample viscosity, which ensures sufficient film thickness at the scale joint contact.

In the presence of a lipid interface, the friction coefficient is drastically diminished in the case of healthy SF. The encapsulation of hyaluronic acid in the lipid vesicles in the healthy case seems to be important to guarantee low wear in more severe situations of lubrication (boundary lubrication)

Thus, if we consider the lubricant capacities of SF, a healthy SF presents the best lubricating properties in both regimens. In the hydrodynamic regimen, the high viscosity of SF ensures good separation between surfaces in contact, despite a high friction coefficient recorded in the absence of lipid interface, whereas in the boundary regimen, low friction coefficients are ensured by the lipid bilayers at the interface which protect against wear at high mechanical load and low relative speed (e.g., a person climbing stairs), in interaction with lipid multilayer vesicles encapsulating HA. In the case of OA, although good hydrodynamic lubrication is ensured, a high degree of wear was recorded as the lipid layers at the interface are torn off after rubbing. This phenomenon is due to the high viscosity of the SF, on one hand, but also to HA present in the medium which sticks to lipid headgroups and destabilizes the lipid layer upon friction. In the case of RA, both regimens are strongly affected: the RA SF suffers from the low viscosity of the hydrodynamic regimen and the lipid layers at the rubbing surfaces in the presence of RA SF are destroyed in the boundary regimen.

The SF is in the meantime an exudate from plasma and a result of active synthesis from synoviocytes. Are SF alterations in OA and RA evidenced in this report a consequence of plasma alterations from inflammatory or metabolic reactions or is the intrinsic synthesis of the synovial vesicles modified? Further analysis of dissociating synoviocyte synthesis from plasma lipid exchange is needed to respond to this question.

Understanding the role of extracellular synovial vesicles in joint physiology is the first step in improving the treatment of joint diseases. For instance, analyzing the composition of the SF before surgery would improve diagnosis and may be an important decisional factor in joint arthroplasty. All these biophysical and biochemical data may be considered for the design of artificial SFs using biomimetic methods [26], for potential therapeutic substitution of defective SFs by an in vitro reconstituted, nanomechanically functional SF, and, as a more immediate goal, for the role of synovial vesicles when analyzing prosthetic materials, which would help to better predict the performances of material in ex-vivo conditions.

## 4. Materials and Methods

### 4.1. Biological Samples and Ethical Statements

The use of pathological human patient biological samples in this study was reviewed by and received approval from the Institutional Review Board of the Lyon University Hospital and the French Ministry of Education and Research (Prof. P. Miossec; authorization N° AC-2010-1164). Healthy biological samples were purchased from LeeBio (Maryland Heights, MO, USA). All biological samples from animals (dog SFs) were collected in full compliance with the Ethical Committee Guidelines and Regulations for Animal Protection of the University Lyon 1 and by the legislation of the European Community (Dr. M.-E. Duclos, DVM; authorization N° 27-41/02-08-2012).

Several types of SFs were analyzed and characterized in terms of structural, biochemical, and tribological properties.

All SF used in this study were acellular (no cells from surrounding tissues were present at the moment of obtaining the samples), non-contaminated with blood, and aseptic.

*Control SF.* As a reference for normal SF, SF samples were collected from 5 healthy male Beagle dogs and 7 healthy human samples collected from healthy volunteers (Leebio, Maryland Heights, MO, USA).

*Representative of degenerative pathology*. SF samples from chronic OA, a degenerative, non-inflammatory type of arthropathy, were collected from 3 patients in the Department of Clinical Immunology and Rheumatology of the Lyon University Hospital Center (Hôpital Edouard Herriot) as previously described [59].

*Representative of inflammatory pathology*. SF samples from RA, an inflammatory disorder that can affect joints were collected from 3 patients in the Department of Clinical Immunology and Rheumatology of the Lyon University Hospital Center (Hôpital Edouard Herriot) as previously described [59].

### 4.2. Phospholipid Extraction and Quantification

PL were extracted from 1 mL SF using the modified Folch method [60] with a mix of chloroform and ethanol (2:1, *v*:*v*). The procedure was repeated twice to make sure that all the lipids were extracted.

The samples were vortexed for about 1 min for each extraction, centrifugated (300× *g*, 25 °C, 5 min) and the organic fraction that contained lipids was recovered and evaporated under a flow of dry nitrogen at room temperature. After total evaporation, the dry residue was recovered with 100 μL extraction solvent. The solution was then stored at −20 °C. The aqueous phase was stored for protein quantification.

The internal lipid standards were added in each sample for sterol esters (ES, 700186), triglycerides (TG, 860903), phosphatidylcholine (PC, 850360), phosphatidylethanolamine (PE, 830756), phosphatidylserine (PS, 840028), sphingomyelin (SM, 860585), and stigmasterol (700062). Standards are made of heptadecanoic acids or margaric acids (C17:0) because these fatty acids are not present in human fats [61] (Avanti Polar Lipids, Alabaster, AL, USA).

### 4.3. Separation of PL, Cholesterol, Sterol Esters, and Triglycerides

An aliquot of 90 μL was deposited on a thin-layer chromatography plate (TCL Silica gen 60 F254, Merck, St Quentin Fallavier, France)) for separation of the different classes of lipids using a solvent mixture of n-hexane, diethyl ether, and acetic acid (80:25:1, *v*:*v*:*v*). Appropriate standards were also deposited on the plate to ensure that separation was achieved and to identify the spot of migration of each lipid class.

Once the elution was completed (about 90 min), the TLC plate was dried under a flow of dry, the sample migration zone was covered with aluminum foil, and the standard zone was sprayed with 0.02% dichlorofluorescein, dried, and visualized under a UV lamp.

As stated before, migration ranges of SF lipids were identified with the help of standard spots. Phospholipids, cholesterol, sterol esters, and triglycerides spots were scraped and collected in tubes, extracted twice from silica plate powder using a small volume of a toluene methanol mixture (1:1, *v*/*v*) then centrifuged (300× *g*, 25 °C, 5 min) to separate silica from the extract.

Final extracts were evaporated again under a flow of dry nitrogen at room temperature. The dry PL extract was recovered in 100 μL chloroform/ethanol (2:1, *v*/*v*). For the quantification of the PL classes, 90 μL of the PL extract was deposited on a TLC plate (Partisil K5 Silica Gel 150 Å, Merk, st Quentin Fallavier, France). Simultaneously, the migration standards for PE, PC, PS/PI/PG, and SM, as described above, were deposited using the capillaries. For the separation of PL by TLC, the solvent system CHCl_3_/MeOH/Methylamine (60/20/5, *v*/*v*/*v*) was used. After migration, the plate was dried under a flow of dry nitrogen at room temperature. The migration standards were revealed with the spray of 0.02% dichlorofluorescein, then visualized under UV. The silica bands corresponding to PE, PC, PS/PI/PG, and SM were scraped and recovered in glass tubes, extracted twice from silica plate powder using a small volume of toluene methanol mix then centrifuged (300× *g*, 25 °C, 5 min) to separate silica from the extract. The solvent was evaporated under a flow of dry nitrogen and the dry extract of each lipid class was recovered with a 500 µL toluene/MeOH mixture (1:1).

### 4.4. Acyl Chain Quantification

Acyl chains were quantified for total lipid extraction, total PL, TG, and esters of sterol, as well as for PC, PE, PS/PI/PG, and SM previously separated as described above by TLC.

A volume of 500 µL of each sample was trans-methylated with 500 μL of a 14% BF_3_/MeOH mixture. The tubes were heated at 100 °C for 90 min (60 min for TG and ES). The reaction was stopped by immersing the tubes in ice and adding 1.5 mL of 10% potassium carbonate. The fatty acid methyl esters obtained were extracted with 2 mL of isooctane Pestipur, then vortexed and centrifuged (5 min, 25 °C, 300 × *g*) and a three-phase separation was obtained: the upper organic phase was recovered in glass tubes and then the solvent was evaporated to dryness under a nitrogen jet. Then, 100 µL of isooctane Pestipur was added to the tubes for gas chromatography. The methyl ester extract was diluted (1/6) with isooctane before injection into the Hewlett Packard gas chromatograph (HP6890, Agilent Technologies, Les Ulis, France). The gas chromatograph was equipped with a flame ionization detector, a programmed temperature injector, and a fused silica capillary column coated with 70% cyanopropyl polysilphénylène siloxane (60 m × 0.22 mm: film thickness 0.25 µm, SGE BPX70, SGE, Trajan Scientific, Victoria, Australia). The initial temperature of the 1 µL-splitless injection was 230 °C. The oven temperature was 50 °C for 2 min, and increased from 50 to 140 °C at a rate of 20 °C min^−1^, then increased to 240 °C at a rate of 2 °C min^−1^ and retained for 5 min. The detector temperature was 250°C under hydrogen flux (45 mL min^−1^); the carrier gas was helium (1 mL min^−1^).

The amount of each lipid class was calculated concerning the amount of C17:0 internal standard.

### 4.5. Protein Quantification

A Bradford assay was used to determine protein concentration in different samples of human synovial fluid. The protein concentration was determined from the absorbance measured at 595 nm using bovine serum albumin (BSA) as standard.

### 4.6. Transmission Electron Microscopy (TEM)

Samples of SF (10 μL aliquots) were adsorbed on carbon-coated copper grids (S160 Carbon Film, 200 Mesh Cu; Agar Scientific, Stansted, Essex, UK) for 5 min at room temperature, and negatively stained with phosphotungstic acid (2% in H_2_O). After draining the excess stain on filter paper, the air-dried specimens were observed under a TOPCON 02B electron microscope (bright-field image mode) operated at 120 kV.

For electron microscopic analysis of microvesicles, an overview of the whole grid was taken, and a maximum number of different pictures showing a minimum of 40 well-resolved vesicles were recorded. Images were analyzed using the ImageJ software, to obtain the distribution of the vesicle size. In the case of a non-spherical shape, the size of a given vesicle was arbitrarily chosen as its maximal diameter.

### 4.7. Rheological Analyses

The rheological measurements were carried out using Rheometer PHYSICA MCR301 (Anton Paar, Graz, Austria) in a cone configuration (ϕ 40 mm) in a stainless steel top at a controlled temperature of 37 °C with a 50 µm air gap, and shear rate varied between 0.01 and 1000 s^−1^.

The contact assembly is surrounded by a water-soaked cloth ensuring a moist saturated atmosphere to prevent evaporation of synovial fluid.

For friction tests, a volume of 0.5 mL of fresh synovial fluid was used.

### 4.8. Tribological Analyses

Tribological measurements were carried out using a homemade bio-tribometer in order to mimic as realistically as possible the operating conditions for a synovial joint under a boundary lubrication regime [25,47,62]. This apparatus allows to visualize the in situ fluorescence contact and to measure the friction coefficient between a hydrophilic soft lens hydroxyethyl methacrylate (HEMA) and a flat borosilicate glass plate, both covered with a PL bilayer constituted of 1-Palmitoyl, 2-oleoyl-phosphatidylcholine (POPC) doped with 2% 1-palmitoyl-2-{6-[(7-nitro-2-1,3-benzoxadiazol-4-yl)amino]hexanoyl}-sn-glycero-3-phosphocholine (NBD-PC) and immerged in SF samples. When submerged in Phosphate-buffered saline (PBS), HEMA presented mechanical properties (elastic and shear modulus) and physico-chemical properties (porosity and hydrophilicity) similar to those of articular cartilage [63]. The evolution of the molecular assemblies of SF was visualized in situ, during friction tests, by fluorescence optical microscopy with NBD-PC as a molecular marker for lipid bilayer alteration [26].

## Data Availability

The data presented in this study are available on request from the corresponding author.

## References

[1] (2006). Standard Test Method for Wear Testing of Polymeric Materials Used in Total Joint Prostheses.

[2] Herbster M., Nizinkovskyi R., Bollmann M., Bartel D., Lohmann C.H., Krüger M., Halle T., Bertrand J. (2021). Synthesis of a Lubricant to Mimic the Biorheological Behavior of Osteoarthritic and Revision Synovial Fluid. Lubricants.

[3] Mazzucco D., McKinley G., Scott R.D., Spector M. (2002). Rheology of joint fluid in total knee arthroplasty patients. J. Orthop. Res..

[4] Mazzucco D., Scott R., Spector M. (2004). Composition of joint fluid in patients undergoing total knee replacement and revision arthroplasty: Correlation with flow properties. Biomaterials.

[5] Dowson D. (2012). Bio-tribology. Faraday Discuss..

[6] Veselack T., Aldebert G., Trunfio-Sfarghiu A.M., Schmid T.M., Laurent M.P., Wimmer M.A. (2018). Phospholipid vesicles in media for tribological studies against live cartilage. Lubricants.

[7] Sava M.-M., Boulocher C., Matei C.I., Munteanu B., Schramme M., Viguier E., Roger T., Berthier Y., Blanchin M.-G., Trunfio-Sfarghiu A.-M. (2013). Structural and tribological study of healthy and biomimetic SF. Comput. Methods Biomech. Biomed. Engin..

[8] Walker P.S., Dowson D., Longfield M.D., Wright V. (1968). “Boosted lubrication” in synovial joints by fluid entrapment and enrichment. Ann. Rheum. Dis..

[9] Wright V., Dowson D., Longfied M.D. (1969). Joint stiffness--its characterisation and significance. Biomed. Eng..

[10] Walker P.S., Dowson D., Longfield M.D., Wright V. (1969). Lubrication of human joints. Ann. Rheum. Dis..

[11] Singh A., Corvelli M., Unterman S.A., Wepasnick K.A., McDonnell P., Elisseeff J.H. (2014). Enhanced lubrication on tissue and biomaterial surfaces through peptide-mediated binding of hyaluronic acid. Nat. Mater..

[12] Ogston A.G., Stanier J.E. (1953). The physiological function of hyaluronic acid in synovial fluid; viscous, elastic and lubricant properties. J. Physiol..

[13] Laurent T.C., Laurent U.B., Fraser J.R. (1995). Functions of hyaluronan. Ann. Rheum. Dis..

[14] Ateshian G.A. (1997). A theoretical formulation for boundary friction in articular cartilage. J. Biomech. Eng..

[15] Sotres J., Arnebrant T. (2013). Experimental Investigations of Biological Lubrication at the Nanoscale: The Cases of Synovial Joints and the Oral Cavity. Lubricants.

[16] Swann D.A., Radin E.L. (1972). The molecular basis of articular lubrication. I. Purification and properties of a lubricating fraction from bovine synovial fluid. J. Biol. Chem..

[17] Swann D.A., Hendren R.B., Radin E.L., Sotman S.L., Duda E.A. (1981). The lubricating activity of synovial fluid glycoproteins. Arthritis Rheum..

[18] Swann D.A., Slayter H.S., Silver F.H. (1981). The molecular structure of lubricating glycoprotein-I, the boundary lubricant for articular cartilage. J. Biol. Chem..

[19] Zappone B., Ruths M., Greene G.W., Jay G.D., Israelachvili J.N. (2007). Adsorption, lubrication, and wear of lubricin on model surfaces: Polymer brush-like behavior of a glycoprotein. Biophys. J..

[20] Klein J. (2006). Molecular mechanisms of synovial joint lubrication. Proc. Inst. Mech. Eng. Part J J. Eng. Tribol..

[21] Han L., Dean D., Ortiz C., Grodzinsky A.J. (2007). Lateral Nanomechanics of Cartilage Aggrecan Macromolecules. Biophys. J..

[22] Goldberg R., Klein J. (2012). Liposomes as lubricants: Beyond drug delivery. Chem. Phys. Lipids.

[23] Higaki H., Murakami T., Nakanishi Y., Miura H., Mawatari T., Inamoto Y. (1998). The lubricating ability of biomembrane models with dipalmitoyl phosphatidylcholine and γ-globulin. Proc. Inst. Mech. Eng. Part H J. Eng. Med..

[24] Hills B.A. (2002). Identity of the joint lubricant. J. Rheumatol..

[25] Trunfio-Sfarghiu A.-M., Berthier Y., Meurisse M.-H., Rieu J.-P. (2008). Role of nanomechanical properties in the tribological performance of phospholipid biomimetic surfaces. Langmuir.

[26] Mirea D.A., Trunfio-Sfarghiu A.-M., Matei C.I., Munteanu B., Piednoir A., Rieu J.P., Blanchin M.-G., Berthier Y. (2013). Role of the biomolecular interactions in the structure and tribological properties of synovial fluid. Tribol. Int..

[27] Matei C.I., Boulocher C., Boulé C., Schramme M., Viguier E., Roger T., Berthier Y., Trunfio-Sfarghiu A.-M., Blanchin M.-G. (2014). Ultrastructural analysis of healthy synovial fluids in three mammalian species. Microsc. Microanal. Off. J. Microsc. Soc. Am. Microbeam Anal. Soc. Microsc. Soc. Canada.

[28] Goldberg R., Schroeder A., Silbert G., Turjeman K., Barenholz Y., Klein J. (2011). Boundary lubricants with exceptionally low friction coefficients based on 2D close-packed phosphatidylcholine liposomes. Adv. Mater..

[29] Goldberg R., Schroeder A., Barenholz Y., Klein J. (2011). Interactions between adsorbed hydrogenated soy phosphatidylcholine (HSPC) vesicles at physiologically high pressures and salt concentrations. Biophys. J..

[30] Watanabe M., Leng C.G., Toriumi H., Hamada Y., Akamatsu N., Ohno S. (2000). Ultrastructural study of upper surface layer in rat articular cartilage by “in vivo cryotechnique” combined with various treatments. Med. Electron Microsc..

[31] Jones C.F., Stoffel K., Ozturk H.E., Stachowiak G.W. (2004). The effect of surface active phospholipids on the lubrication of osteoarthritic sheep knee joints: Wear. Tribol. Lett..

[32] Petelska A.D., Kazimierska-Drobny K., Janicka K., Majewski T., Urbaniak W. (2019). Understanding the Unique Role of Phospholipids in the Lubrication of Natural Joints: An Interfacial Tension Study. Coatings.

[33] Theobald P.S., Byrne C., Oldfield S.F., Dowson D., Benjamin M., Dent C., Pugh N., Nokea L.D.M. (2007). Lubrication regime of the contact between fat and bone in bovine tissue. Proc. Inst. Mech. Eng. Part H J. Eng. Med..

[34] Kawano T., Miura H., Mawatari T., Moro-Oka T., Nakanishi Y., Higaki H., Iwamoto Y. (2003). Mechanical effects of the intraarticular administration of high molecular weight hyaluronic acid plus phospholipid on synovial joint lubrication and prevention of articular cartilage degeneration in experimental osteoarthritis. Arthritis Rheum..

[35] Forsey R.W., Fisher J., Thompson J., Stone M.H., Bell C., Ingham E. (2006). The effect of hyaluronic acid and phospholipid based lubricants on friction within a human cartilage damage model. Biomaterials.

[36] Seror J., Zhu L., Goldberg R., Day A.J., Klein J. (2015). Supramolecular synergy in the boundary lubrication of synovial joints. Nat. Commun..

[37] Schwarz I.M., Hills B.A. (1998). Surface-active phospholipid as the lubricating component of lubricin. Br. J. Rheumatol..

[38] Schmidt T.A., Sah R.L. (2007). Effect of synovial fluid on boundary lubrication of articular cartilage. Osteoarthr. Cartil..

[39] Chen D., Yu H., Zhang Y., Huang Y. (2022). Metabolomic analysis of extracellular vesicles from human synovial fluids. Microchem. J..

[40] Sarma A.V., Powell G.L., LaBerge M. (2001). Phospholipid composition of articular cartilage boundary lubricant. J. Orthop. Res..

[41] Kosinska M.K., Liebisch G., Lochnit G., Wilhelm J., Klein H., Kaesser U., Lasczkowski G., Rickert M., Schmitz G., Steinmeyer J. (2013). A lipidomic study of phospholipid classes and species in human synovial fluid. Arthritis Rheum..

[42] Kosinska M.K., Mastbergen S.C., Liebisch G., Wilhelm J., Dettmeyer R.B., Ishaque B., Rickert M., Schmitz G., Lafeber F., Steinmeyer J. (2016). Comparative lipidomic analysis of synovial fluid in human and canine osteoarthritis. Osteoarthr. Cartil..

[43] Kosinska M.K., Liebisch G., Lochnit G., Wilhelm J., Klein H., Kaesser U., Lasczkowski G., Rickert M., Schmitz G., Steinmeyer J. (2014). Sphingolipids in human synovial fluid--a lipidomic study. PLoS ONE.

[44] Kosinska M.K., Ludwig T.E., Liebisch G., Zhang R., Siebert H.-C., Wilhelm J., Kaesser U., Dettmeyer R.B., Klein H., Ishaque B. (2015). Articular Joint Lubricants during Osteoarthritis and Rheumatoid Arthritis Display Altered Levels and Molecular Species. PLoS ONE.

[45] Mustonen A.M., Käkelä R., Joukainen A., Lehenkari P., Jaroma A., Kääriäinen T., Kröger H., Paakkonen T., Sihvo S.P., Nieminen P. (2021). Synovial fluid fatty acid profiles are differently altered by inflammatory joint pathologies in the shoulder and knee joints. Biology.

[46] Sivan S., Schroeder A., Verberne G., Merkher Y., Diminsky D., Priev A., Maroudas A., Halperin G., Nitzan D., Etsion I. (2010). Liposomes Act as Effective Biolubricants for Friction Reduction in Human Synovial Joints. Langmuir.

[47] Trunfio-Sfarghiu A.-M., Berthier Y., Meurisse M.-H., Rieu J.-P., Dowson D. (2007). Multiscale analysis of the tribological role of the molecular assemblies of synovial fluid: Case of a healthy joint and implants. Tribol. Int..

[48] Sorkin R., Kampf N., Zhu L., Klein J. (2016). Hydration lubrication and shear-induced self-healing of lipid bilayer boundary lubricants in phosphatidylcholine dispersions. Soft Matter.

[49] Eiser E., Klein J. (2007). The Effect of Mobile Polymers on the Normal and Shear Forces between Polymer Brushes. Macromolecules.

[50] van Meer G. (2005). Cellular lipidomics. EMBO J..

[51] Clarke C.J., Truong T.G., Hannun Y.A. (2007). Role for neutral sphingomyelinase-2 in tumor necrosis factor α-stimulated expression of vascular cell adhesion molecule-1 (VCAM) and intercellular adhesion molecule-1 (ICAM) in lung epithelial cells: p38 MAPK is an upstream regulator of nSMase2. J. Biol. Chem..

[52] Maceyka M., Spiegel S. (2014). Sphingolipid metabolites in inflammatory disease. Nature.

[53] Schurz J., Ribitsch V. (1987). Rheology of synovial fluid. Biorheology.

[54] Hills B.A., Crawford R.W. (2003). Normal and prosthetic synovial joints are lubricated by surface-active phospholipid: A hypothesis. J. Arthroplasty.

[55] Hills B.A. (1989). Oligolamellar lubrication of joints by surface active phospholipid. J. Rheumatol..

[56] Pawlak Z., Urbaniak W., Oloyede A. (2011). The relationship between friction and wettability in aqueous environment. Wear.

[57] Corneci M.C., Dekkiche F., Trunfio-Sfarghiu A.M., Meurisse M.H., Berthier Y., Rieu J.P. (2011). Tribological properties of fluid phase phospholipid bilayers. Tribol. Int..

[58] Mustonen A.M., Nieminen P. (2021). Fatty Acids and Oxylipins in Osteoarthritis and Rheumatoid Arthritis—A Complex Field with Significant Potential for Future Treatments. Curr. Rheumatol. Rep..

[59] Toh M.L., Gonzales G., Koenders M.I., Tournadre A., Boyle D., Lubberts E., Zhou Y., Firestein G.S., van den Berg W.B., Miossec P. (2010). Role of interleukin 17 in arthritis chronicity through survival of synoviocytes via regulation of synoviolin expression. PLoS ONE.

[60] Folch J., Lees M., Sloane Sranley G.H. (1957). A simple method for the isolation and purification of total lipides from animal tissues. J. Biol. Chem..

[61] Martins-Noguerol R., Moreno-Pérez A.J., Acket S., Makni S., Garcés R., Troncoso-Ponce A., Salas J.J., Thomasset B., Martínez-Force E. (2019). Lipidomic Analysis of Plastidial Octanoyltransferase Mutants of Arabidopsis thaliana. Metabolites.

[62] Dekkiche F., Corneci M.C., Trunfio-Sfarghiu A.-M., Munteanu B., Berthier Y., Kaabar W., Rieu J.-P. (2010). Stability and tribological performances of fluid phospholipid bilayers: Effect of buffer and ions. Colloids Surf. B. Biointerfaces.

[63] Bostan L., Trunfio-Sfarghiu A.M., Verestiuc L., Popa M.I., Munteanu F., Rieu J.P., Berthier Y. (2012). Mechanical and tribological properties of poly(hydroxyethyl methacrylate) hydrogels as articular cartilage substitutes. Tribol. Int..

